# Correlates of Social Isolation Among Community-Dwelling Older Adults During the COVID-19 Pandemic

**DOI:** 10.3389/fpubh.2021.702965

**Published:** 2021-12-10

**Authors:** Omolola E. Adepoju, Minji Chae, LeChauncy Woodard, Kendra L. Smith, Luz Herrera, Daikwon Han, Daniel L. Howard, Jessica Dobbins, Marcia Ory

**Affiliations:** ^1^Department of Health Systems and Population Health Sciences, University of Houston College of Medicine, Houston, TX, United States; ^2^Humana Integrated Health System Sciences Institute, University of Houston College of Medicine, Houston, TX, United States; ^3^Texas A&M University School of Law, Fort Worth, TX, United States; ^4^Department of Epidemiology and Biostatistics, Texas A&M School of Public Health, College Station, TX, United States; ^5^Department of Psychological and Brain Sciences, Texas A&M University, College Station, TX, United States; ^6^Office of Health Affairs and Advocacy, Humana Inc., Louisville, KY, United States; ^7^Center for Population Health and Aging, Texas A&M School of Public Health, College Station, TX, United States; ^8^Department of Environmental and Occupational Health, Texas A&M School of Public Health, College Station, TX, United States

**Keywords:** social isolation, aging, COVID-19, racial/ethnic differences, loneliness

## Abstract

**Background:** The past year has severely curtailed social interactions among older adults given their high rates of COVID-19 morbidity and mortality. This study examined social, behavioral, and medical correlates of social isolation among community-dwelling older adults during the COVID-19 pandemic and stratified findings to explore unique differences in two typically neglected populations, African American and Hispanic older adults.

**Methods:** Working with community-based organizations and senior living centers, the research team administered a survey to older adults 55 years of age and older (*n* = 575). The survey assessed COVID-19 prevention behaviors, medical conditions, and lived experiences, including feelings of social isolation, in the target population. Responses to a previously validated social isolation question informed a dichotomous social isolation dependent variable. Multivariable logistic regression was used to adjust for sociodemographic characteristics, medical conditions, unmet caregiving needs, and COVID-19 prevention behaviors. Results from the regression model were stratified by race/ethnicity to examine correlates of social isolation in African American and Hispanic older adults, separately.

**Results:** Overall, female sex and a higher level of education were also positively associated with social isolation (OR = 2.46, *p* = 0.04; OR = 5.49, *p* = 0.02) while having insurance exhibited an inverse relationship (OR = 0.25, *p* = 0.03). Unmet caregiving needs were strongly associated with social isolation (OR = 6.41, *p* < 0.001) as was having any chronic conditions (OR = 2.99, *p* = 0.02). Diabetes was the single strongest chronic condition predictor of social isolation. Among minority older adults, a different pattern emerged. For Hispanic older adults, language, unmet caregiving needs, and social distancing were strongly associated with social isolation; while unmet caregiving needs, having 1+ chronic conditions and adhering to social distancing guidelines were significant predictors in African American older adults.

**Conclusion:** These findings suggest that social isolation affects older adults in a myriad of ways and support the need for culturally sensitive initiatives to mitigate the effect of social isolation in these vulnerable populations.

## Introduction

Remaining connected in an era of social distancing is paramount to maintaining health and well-being when life as we know it has been upended, daily routines and activities canceled, and generations of families have been separated to protect the health of those most vulnerable. Since the onset of COVID-19, social connections and engagement with older adults have been severely curtailed given the very high rates of COVID-19 morbidity/mortality in this population. Global estimates suggest that COVID-19 related fatality is over-represented among older adults (1). Recent statistics in the U.S. illustrate this with ~75% of all adults hospitalized for COVID-19 being at least 50 years old, and more than 80% of COVID-19 fatalities among those aged 65 years and older (2, 3). While preventive and management measures are important to mitigate the spread of COVID-19, strategies like social distancing can have devastating effects on older adults who are already at risk for social isolation and loneliness.

In the U.S., nearly 30% of the 46 million community-dwelling older adults live alone (4). Older adults living alone are more likely to be poor, especially with advancing age, and to report feelings of loneliness (60% of those 75+) and isolation (4). Socially isolated older adults have a 64 percent risk of developing clinical dementia and a 29% risk of premature death and are more likely to experience psychological distress, even after accounting for socioeconomic factors (4). Recent studies examining the impact of the pandemic on disease management patterns associated the absence of social support with decreased positive self-management behaviors, such as physical activity, dietary modifications, glucose monitoring, and smoking cessation (5, 6). In the early months of the population lockdowns, individuals who had low levels of social capital, social support, and neighborhood relationships experienced more depression, anxiety, stress, and poor sleep quality due to the lockdown (7). Others have equated the impact of social isolation on health status and mortality to the impact of smoking, obesity, and lack of exercise (8).

While medical consequences of COVID-19 are often highlighted, the public health impacts are equally concerning. Compounded by the pandemic-induced fall-out with the loss of traditional sources of support, many older adults struggle to access food, pay their bills, and access community resources (9). The “social distancing” mandates have only amplified existing high levels of social isolation and loneliness (10), and fears about COVID-19 and impacts on daily life have increased depression and anxiety (11). Minority and ethnic populations have also been hit especially hard in terms of experiencing both negative medical and social impacts (12). For many minority older adults, who tend to rely on family and community support or caregivers, the effects of social isolation likely differ from the effects observed in the general older adult population. For example, higher socioeconomic status (SES) and white families are more likely to provide financial and emotional support while lower SES, Black, and Latino families tend to provide practical help and co-reside in multigenerational households (13).

In response to this public health crisis, national, state, and local efforts have begun to raise awareness about the health risks associated with social isolation and loneliness and implement preventative mitigation measures. For example, experts recommend that older adults talk with friends and family regularly, keep a healthy lifestyle, and get outdoors as much as possible—keys to maintaining good physical and mental health—to combat loneliness and isolation (14). Beyond that, many state and local agencies are utilizing technology to foster virtual connections, while others have created hotlines and dedicated resources toward reaching, engaging, and supporting older individuals. Some states, including, but not limited to Texas, have created 24/7 COVID-19 Mental Health Support Lines to talk with a mental health professional while others have developed interventions ranging from wellness check-in programs to food delivery and robotic pets (15, 16). There have also been a plethora of media campaigns, well-being webinars, and mobile applications to provide practical tips on coping strategies (17, 18).

Despite these resources, aging experts contend that the fight against social isolation remains in its infancy (19) and the need for culturally sensitive approaches to address social isolation in unique racial/ethnic groups remains unaddressed. To address these research and practice gaps, this study assesses social isolation among community-dwelling older adults during the COVID-19 pandemic and stratifies findings to explore unique differences in two typically neglected populations, African American and Hispanic older adults. This study examines social, behavioral, and medical correlates of social isolation during the COVID-19 pandemic among older adults living in a large metropolitan area. Studies of this nature are helpful to identify especially vulnerable populations and to guide culturally appropriate intervention strategies.

## Methods

### Data

Working with community-based organizations and senior living centers, an electronic survey was disseminated to older adults 55 years of age and older, in the Houston metroplex, between 11/2020 and 01/2021. The survey assessed COVID-19 prevention behaviors, medical conditions, and lived experiences, including feelings of social isolation, in the target population. The survey included previously validated questions, drawing from the Accountable Health Communities Health-Related Social Needs (AHC HRSN) screening tool, and included some new questions about COVID-19 behaviors and pandemic response. A subset of authors (OA, LW, and LH) assessed the survey constructs for face validity and cultural relevancy.

### Measurement

All items were self-reported during online survey administration, which took ~10 min to complete. Responses to the AHC HRSN validated question “How often do you feel lonely or isolated from those around you?” were used to create a dichotomous dependent variable, where “Never,” “Rarely” and “Sometimes” represented 0 (not socially isolated) while “Often” and “Always” represented 1 (socially isolated). The rationale for dichotomizing is based on earlier work on social isolation that classifies adults responding “Often” and “Always” as socially isolated (20). The main independent variable, family/community support to meet caregiving needs, was a binary indicator based on responses to the validated AHC HRSN screening question “If for any reason you need help with day-to-day activities such as bathing, preparing meals, shopping, managing finances, etc., do you get the help you need?” Participants who indicated they could “use a little more help,” or they needed “a lot more help” were flagged as 1 while those who indicated they “don't need any help” or “get all the help I need” were flagged as 0. This variable will be subsequently referred to as unmet *caregiving needs*. Other covariates included sociodemographic characteristics (e.g., age, sex, race/ethnicity, education level, income, insurance status), medical conditions (e.g., various chronic conditions, positive COVID test for self or close family member), COVID-19 prevention behaviors (e.g., social distancing), and social needs (e.g., disability, Supplemental Security Income, Social Security, debt, income instability, trouble paying for medication, loss of transportation). All variables are theoretically grounded and were used for the full and stratified regression models.

### Analysis

Descriptive analyses employing frequencies and proportions were used to describe patient demographic characteristics. Chi-square tests were used to assess independent bivariate associations between respondent characteristics and social isolation. Multivariable logistic regression examined the strength of the relationships, adjusting for sociodemographic characteristics, medical conditions, COVID-19 prevention behaviors, and social needs. Results from the regression model were stratified by race/ethnicity to examine correlates of social isolation in African American older adults and Hispanic older adults, separately. This study was approved by an independent institutional review board in October 2020 (IRB ID: STUDY00002584). Using a 2-tailed α = 0.05, we were sufficiently powered to detect a minimum expected difference of 10% in the proportion of older adults reporting social isolation. All data management and analyses were performed using Stata 16.1. All statistical tests were 2-sided, and findings were considered statistically significant at *p* < 0.05.

## Results

### Sample Characteristics

Overall, the sample contained 575 survey responses. Survey respondents comprised 24% males, 39% Hispanics, and 51% Black or African Americans. Thirty-four percentage of the sample represented older adults between 55 and 64 years, 42% represented older adults between the ages of 65 and 74, and 25% represented adults 75 years and older. Overall, 24% of respondents were uninsured, and 43% had an annual income < $25,000 while nearly 20% had an annual income of $75,000 or higher. Regarding education level, 20% of the respondents did not have a high school diploma, 26% had a high school diploma or GED, 27% had some college education, and 27% were college educated, having bachelor's or graduate degrees. Seventy-two percent of the sample indicated English as their primary language while 61% were homeowners. Chronic condition and disease burden included heart disease (19%), chronic lung disease (7%), diabetes (20%), psychological/psychiatric conditions (27%), Rheumatoid Arthritis, Lupus, or other autoimmune conditions (24%), and stroke (5%). Nineteen percent of the sample reported limited activity due to health conditions, while 13% have health problems that require special equipment. In responding to their caregiving needs, 28% indicated they had caregiving needs. Over half of respondents had tested positive for COVID-19 or had a close family member who had tested positive in the past month. Only 47% indicated they were practicing social distancing. Approximately 10% perceived themselves as socially isolated.

### Bivariate Analysis

Table 1 shows the bivariate associations of survey responses by perceived social isolation. Social isolation was significantly associated with having a chronic disease; 80% of those who reported social isolation had one or more of the six chronic conditions that were assessed vs. 56% among those who were not socially isolated (*p* < 0.001). Having diabetes was strongly associated with social isolation (33% of those who reported social isolation vs. 18% among those not socially isolated, *p* < 0.01). Experiencing limited activity due to health conditions was also significantly related to social isolation (28% of those who reported social isolation vs. 17% among those not socially isolated, *p* = 0.04). Among those who were socially isolated, 55% had caregiving needs vs. 24% among those who were not socially isolated (*p* < 0.001). None of the other independent bivariate relationships attained statistical significance. For example, in bivariate analyses, there were no differences in perceived social isolation by minority status.

**Table 1:** Bivariate associations of survey respondents by social connectedness.

**Variables**	**Total (*n* = 575)**	**Social Isolation**		
		**Not Socially Isolated (*N* = 515)**	**Socially Isolated (*N* = 60)**	***p***
	***N* (%)**	***N* (%)**	***N* (%)**	
Age				0.078
55–64	193 (33.57)	175 (33.98)	18 (30.00)	
65–74	239 (41.57)	219 (42.52)	20 (33.33)	
≥75	143 (24.87)	121 (23.50)	22 (36.67)	
Race/ethnicity				0.711
White/non-minority others	53 (9.38)	47 (9.27)	6 (10.30)	
Black or African American	290 (51.33)	257 (50.69)	33 (56.90)	
Hispanic	222 (39.29)	203 (40.04)	19 (32.76)	
Gender				0.156
Female	429 (75.93)	379 (75.05)	50 (83.33)	
Male	136 (24.07)	126 (24.95)	10 (16.67)	
Insurance				0.230
No insurance	137 (23.83)	125 (24.27)	12 (20.00)	
Medicaid	57 (9.91)	49 (9.51)	8 (13.33)	
Medicare	258 (44.87)	226 (43.88)	32 (53.33)	
Private Insurance	123 (21.39)	115 (22.33)	8 (13.33)	
Income				0.080
$0–24,999	217 (42.80)	195 (43.24)	22 (39.29)	
$25,000–74,999	192 (37.87)	164 (36.36)	28 (50.00)	
≥$75,000	98 (19.33)	92 (20.40)	6 (10.71)	
Highest level of education				0.661
No highschool	111 (20.33)	102 (20.90)	9 (15.52)	
GED/highschool	141 (25.82)	127 (26.02)	14 (24.14)	
Some college	146 (26.74)	130 (26.64)	16 (27.59)	
College (bachelors, graduate)	148 (27.11)	129 (26.43)	19 (32.76)	
Own home				0.930
No	227 (39.48)	203 (39.42)	24 (40.00)	
Yes	348 (60.52)	312 (60.58)	36 (60.00)	
Primary language				0.764
Others	163 (28.35)	145 (28.16)	18 (30.00)	
English	412 (71.65)	370 (71.84)	42 (70.00)	
Chronic conditions				
Any chronic disease	338 (58.78)	290 (56.31)	48 (80.00)	<0.001
Heart disease	112 (19.48)	96 (18.64)	16 (26.67)	0.137
Chronic lung disease	42 (7.30)	38 (7.38)	4 (6.67)	0.841
Diabetes	114 (19.83)	94 (18.25)	20 (33.33)	0.006
Psychological/psychiatric conditions	158 (27.48)	140 (27.18)	18 (30.00)	0.644
RA^*^, Lupus, autoimmune disorders	137 (23.83)	122 (23.69)	15 (25.00)	0.822
Stroke	26 (4.52)	22 (4.27)	4 (6.67)	0.398
Limited activity due to condition				0.041
No	468 (81.39)	425 (82.52)	43 (71.67)	
Yes	107 (18.61)	90 (17.48)	17 (28.33)	
Health problem(s) that require equipment				0.166
No	502 (87.30)	453 (87.96)	49 (81.67)	
Yes	73 (12.70)	62 (12.04)	11 (18.33)	
Caregiving needs				<0.001
No caregiving needs	416 (72.35)	389 (75.53)	27 (45.00)	
Have caregiving needs	159 (27.65)	126 (24.47)	33 (55.00)	
Positive COVID test (self or close family)				0.350
No	282 (49.04)	256 (49.71)	26 (43.33)	
Yes	293 (50.96)	259 (50.29)	34 (56.67)	
Social distancing				0.187
Not Practicing social distancing	305 (53.04)	278 (53.98)	27 (45.00)	
Practicing social distancing	270 (46.96)	237 (46.02)	33 (55.00)	

* *RA, rheumatoid arthritis*.

### Multivariate Analysis

The logistic regression results are shown in Table 2. Females were marginally more likely to be socially isolated (OR = 2.46; *p* = 0.04). Compared to older adults who indicated they have no GED/high school diploma, those who were college educated were 2.6 times more likely to report feelings of socially isolation during the pandemic (*p* = 0.05). Respondents who reported having private insurance were less likely to report feeling of social isolation (OR = 0.25, *p* = 0.03). Those who had unmet caregiving needs were 6.4 times more likely to report social isolation than those who reported needing no help (*p* < 0.001). Additionally, persons with one or more chronic conditions were 2.9 times more likely to report social isolation than persons without any chronic conditions (*p* = 0.02). When the research team considered chronic diseases separately, diabetes was the only statistically significant medical condition associated with social isolation. None of the other covariates attained statistical significance.

**Table 2:** Multivariable regression model of the relationship between social isolation and respondent characteristics (*n* = 575).

**Variables**	**Odds ratio**	**95%CI**		***P***
**Age**				
55–64	Ref.			
65–74	1.57	0.64	−3.63	0.337
≥75	1.88	0.72	−4.84	0.196
**Race/ethnicity**				
Hispanic/LatinX	Ref.			
White/non-minority others	1.55	0.55	−3.34	0.487
Black or African American	1.16	0.47	−2.56	0.795
**Gender**				
Male	Ref.			
Female	2.46	1.06	5.80	0.038
**Insurance**				
No insurance	Ref.			
Medicaid	1.11	0.35	−3.69	0.827
Medicare	0.55	0.19	−1.58	0.265
Private Insurance	0.25	0.07	−0.94	0.034
**Income**				
$0–24,999	Ref.			
$25,000–74,999	1.09	0.56	−2.47	0.623
≥$75,000	0.61	0.22	−1.94	0.414
**Highest level of education**				
No high school	Ref.			
GED/high school	1.45	0.49	−4.30	0.506
Some college/technical/vocational	1.40	0.42	−4.62	0.538
College (bachelor's, graduate)	2.58	0.97	−11.19	0.053
**Own home**				
No	Ref.			
Yes	1.21	0.59	−2.69	0.606
**Caregiver needs**				
Not met	Ref.			
Met	6.41	3.03	−10.65	<0.001
**Positive COVID test (self or close family)**				
No	Ref.			
Yes	1.45	0.71	−2.98	0.247
**English**				
No	Ref.			
Yes	0.47	0.19	−1.19	0.112
**Limited activity**				
No	Ref.			
Yes	1.26	0.52	−3.03	0.604
**Health problems requiring special equipment**				
No	Ref.			
Yes	0.72	0.24	−2.08	0.544
**Any chronic disease**				
No	Ref.			
Yes	2.99	1.24	−7.19	0.015
**Social distancing**				
Not practicing social distancing	Ref.			
Practicing social distancing	1.84	0.95	−3.71	0.070
Log Likelihood	−135.95			
Pseudo *R*-squared	0.2110			

### Cohort Sub-analyses

Results of the race/ethnicity stratified model are presented in Table 3. African Americans (*n* = 290) who had unmet caregiving needs were 3.9 times more likely to report social isolation than those who reported needing no help (*p* = 0.01). African Americans who reported English as their primary language were significantly less likely to be socially isolated (OR = 0.17, *p* = 0.04), while those who had one or more chronic conditions were more likely to report social isolation (OR = 7.69; *p* = 0.05). African Americans who indicated they were practicing social distancing were also more likely to report feelings of social isolation (OR = 1.41, *p* = 0.04).

**Table 3:** Multivariable regression model of the relationship between social isolation and respondent characteristics: correlates of social isolation in African Americans and Hispanic Older Adults.

	**African Americans**	***P***	**Hispanic/LatinX**	***P***
	***n* = 290**		***n* = 222**	
	**Odds Ratio**		**Odds Ratio**	
**Age**				
<65	Ref.		Ref.	
65–74	1.03	0.96	3.15	0.18
≥75	1.50	0.55	6.49	0.07
**Gender**				
Male	Ref.		Ref.	
Female	3.50	0.06	1.37	0.71
**Insurance**				
No insurance	Ref.		Ref.	
Medicaid	1.48	0.74	0.34	0.30
Medicare	0.85	0.86	0.46	0.03
Private Insurance	0.29	0.22	0.74	0.80
**Income**				
$0–24,999	Ref.		Ref.	
$25,000–74,999	0.65	0.44	2.10	0.35
≥$75,000	0.57	0.46	–	–
**Highest level of education**				
No highschool	Ref		Ref.	
GED/highschool	0.20	0.26	2.46	0.26
Some college/technical/vocational	0.58	0.67	1.28	0.83
College (bachelor's, graduate)	1.38	0.76	–	–
**Own home**				
No	Ref.		Ref.	
Yes	2.06	0.28	0.98	0.98
**Caregiver needs**				
Not met	Ref.		Ref.	
Met	3.94	0.01	11.66	0.002
**Positive COVID test (self or close family)**				
No	Ref.		Ref.	
Yes	1.46	0.49	1.18	0.81
**English**				
No	Ref.		Ref.	
Yes	0.17	0.04	0.70	0.05
**Limited activity**				
No	Ref.		Ref.	
Yes	1.12	0.84	4.77	0.10
**Health problems requiring special equipment**				
No	Ref.			
Yes	1.08	0.06	0.51	0.66
**Any chronic disease**				
No	Ref.		Ref.	
Yes	7.69	0.05	2.80	0.17
**Social distancing**				
Not practicing social distancing	Ref.		Ref.	
Practicing social distancing	1.41	0.04	0.51	0.66
Log Likelihood	−75.81		−34.87	
Pseudo *r*-squared	0.2305		0.2923	

Comparatively, Hispanic older adults (*n* = 222) who reported having Medicare insurance were significantly less likely to be socially isolated (OR = 0.46, *p* = 0.03). As with the African American respondents, Hispanic older adults who had unmet caregiving needs were 11.6 times more likely to report social isolation than those who reported needing no help (*p* = 0.002). Primary language also had a significant effect so that Hispanic older adults who reported English as their primary language were less likely to be socially isolated (OR = 0.70, *p* = 0.05).

## Discussion

This study assessed the correlates of social isolation among community-dwelling older adults and explored unique differences for African American and Hispanic older adults, providing a rare glimpse into COVID-19 impacts among populations typically seen as more under-resourced. In the total older population (55 and older), we found no significant differences by minority/ethnic status in either bivariate or multivariate analyses predicting social isolation. However, females were more likely to report a feeling of social isolation, as were adults with advanced education degrees, those with unmet caregiving needs, and those with 1+ chronic conditions. Being privately insured was protective such that those who reported having private insurance were less likely to report social isolation. Workforce participation may help explain this relationship.

Comparatively, among African American adults, gender, education, and insurance status were no longer significant, while those with unmet caregiving needs, those with 1+ chronic conditions, and those who indicated they adhere to social distancing guidelines were more likely to be socially isolated. In the Hispanic population, language, unmet caregiving needs, and social distancing were significantly associated with social isolation. These findings are poignant in disputing the one-size-fits all notion of social isolation impacts among older adults and support the need for culturally sensitive initiatives to mitigate the impact of social isolation in these vulnerable populations.

In the wake of the COVID-19 pandemic, social distancing and self-isolation measures were implemented in efforts to reduce the transmission of the disease. African American and Hispanic seniors who reported adhering to these guidelines were more likely to report feelings of social isolation. While these strategies are necessary, they pose potential threats to the physical and mental health of those following such precautions, particularly because these minority populations rely more on family and community support. Many older adults, particularly within the African American and Latinx communities, tend to have less knowledge about navigating newer technologies that provide information on how to manage social distancing and serve as an outlet to stay connected with friends and family when people are unable to meet in person (21). Minority populations are also more reliant on smaller social networks that are associated with places where they congregate, such as religious organizations, for psychological and social support, and when these avenues are taken away, it is more difficult for them to avoid isolation (21). Because African Americans and Hispanic Americans were disproportionately affected by COVID deaths, the loss of social network and the experience of grief could further exacerbate isolation in this vulnerable population (21). Individuals within the Latinx community who also have low English proficiency may have less access to linguistically relevant information about COVID-19, self-isolation, and keeping their loved ones safe (22).

For those with caregiving needs, such as bathing, preparing meals, managing their finances, and other day-to-day activities, our findings reveal a strong positive association with COVID-induced social isolation, across all racial/ethnic subgroups. This aligns with the notion that adults who are functionally dependent on family members or other forms of community support are at a higher risk for isolation because they rely on these social and community connections. Minority older adults who had extended-family caregivers and lived in a multi-generational home still experienced high levels of loneliness if they did not feel that they were contributing to their surrounding community (23). Unfortunately, it appears that COVID-19 continues to negatively impact those who are most vulnerable physically, and this pattern remains strong across racial/ethnic population sub-groups. Strategies to mitigate the impact of social isolation need to focus on these vulnerable adults.

We also observe that adults with chronic conditions, particularly diabetes, were significantly more likely to feel socially isolated during the pandemic. This finding aligns with previous studies that show an association between having diabetes and feeling socially isolated (24). Older adults suffering from chronic conditions while living alone experienced extremely high levels of loneliness compared to other groups because they self-isolated more, in part due to the greater perceived threat of the virus (25). Perhaps due to the fear of contracting COVID-19, older adults with chronic conditions adhered to public health guidelines more strictly than others, consequently contributing to the elevated levels of perceived social isolation and loneliness. For older adults with diabetes, Ida et al. suggests that social isolation is related to increased glycemic fluctuations, implying that there may be a link between social isolation and poor diabetes management (26). Older adults with chronic conditions, particularly diabetes, are most likely to benefit from interventions designed to reduce social isolation.

Although our findings suggest that social isolation differentially impacts older adults, the implications need to be framed within the context of the COVID-19 pandemic. For instance, while we found that overall, those with graduate degrees were more likely to feel socially isolated, this finding did not hold in the African American or Hispanic sub-cohorts. This overall finding differs from previous studies that show lower education levels are significantly associated with social isolation (27, 28). However, we must take into consideration the advantages of having a graduate degree in the setting of a pandemic. Those with graduate or professional degrees likely have the privilege of working remotely, which allows them to better abide by the stay-at-home mandates put in place. While they are granted the opportunity to limit their exposure to the virus, working remotely may contribute to the perception of social isolation.

While these findings are informative, this study is not without limitations. This study employs a cross-sectional survey in the fifth largest metropolitan area in the U.S. and hence, findings may not be generalizable to other areas of the country. It is also unclear whether these results will persist when COVID-19 transmission rates are lower, so findings may not be generalizable to other time periods. Lastly, these findings may not be generalizable to persons <55 years of age. Despite these potential limitations, our findings are important and shed additional light on the correlates of social isolation in older adults, and how these findings vary for minority populations. The COVID-19 pandemic has changed how people live and its impact has been broad. While social restrictions are crucial during the pandemic, it is important to recognize the populations most affected by COVID-19-induced social isolation and loneliness. These communities face an increased risk of potential mental and physical consequences. Evaluations such as the present study allow us to address the impact of social isolation during the post-pandemic period to ensure there are minimal lasting effects of COVID-19 on physical and mental health.

## Data Availability Statement

The datasets presented in this article are not readily available because within the IRB submission, we included a statement that raw data will not leave the University of Houston. Requests to access the datasets should be directed to Omolola E. Adepoju, oadepoju@uh.edu.

## Ethics Statement

The studies involving human participants were reviewed and approved by University of Houston IRB. The patients/participants provided their written informed consent to participate in this study.

## Author Contributions

OA conceptualized the study, conducted the data analysis, and wrote the initial draft of the manuscript. MC assisted with data collection, analysis, and manuscript writing. LW, KS, LH, DH, and DLH participated in fund acquisition, study design, and reviewed versions of the manuscript. JD assisted with manuscript preparation and revisions. MO helped to frame the study and edited multiple versions. All authors contributed to the article and approved the submitted version.

## Funding

This work was supported by the Walmart Foundation for the Study on Disaster Response and Recovery among Minority Older Adults in Houston, Texas, from the Effects of Pandemics (Award Number # 62322251).

## Conflict of Interest

JD is employed with Humana Inc. The remaining authors declare that the research was conducted in the absence of any commercial or financial relationships that could be construed as a potential conflict of interest.

## Publisher's Note

All claims expressed in this article are solely those of the authors and do not necessarily represent those of their affiliated organizations, or those of the publisher, the editors and the reviewers. Any product that may be evaluated in this article, or claim that may be made by its manufacturer, is not guaranteed or endorsed by the publisher.
